# Oceanic thermal structure mediates dive sequences in a foraging seabird

**DOI:** 10.1002/ece3.6393

**Published:** 2020-05-24

**Authors:** Xavier Meyer, Andrew J. J. MacIntosh, Andre Chiaradia, Akiko Kato, Francisco Ramírez, Cédric Sueur, Yan Ropert‐Coudert

**Affiliations:** ^1^ CNRS IPHC UMR7178 Université de Strasbourg Strasbourg France; ^2^ Kyoto University Primate Research Institute Inuyama Japan; ^3^ Conservation Department Phillip Island Nature Parks Cowes Vic Australia; ^4^ Centre d'Etudes Biologiques de Chizé CNRS UMR 7372 Université de La Rochelle Villiers‐en‐Bois France; ^5^ Departament de Biologia Evolutiva Ecologia i Ciènces Ambientals Universitat de Barcelona Barcelona Spain

**Keywords:** behavioral complexity, *Eudyptula minor*, foraging behavior, fractal analysis, little penguin, sea surface temperature, thermocline

## Abstract

1. Changes in marine ecosystems are easier to detect in upper‐level predators, like seabirds, which integrate trophic interactions throughout the food web.
2. Here, we examined whether diving parameters and complexity in the temporal organization of diving behavior of little penguins (*Eudyptula minor*) are influenced by sea surface temperature (SST), water stratification, and wind speed—three oceanographic features influencing prey abundance and distribution in the water column.
3. Using fractal time series analysis, we found that foraging complexity, expressed as the degree of long‐range correlations or memory in the dive series, was associated with SST and water stratification throughout the breeding season, but not with wind speed. Little penguins foraging in warmer/more‐stratified waters exhibited greater determinism (memory) in foraging sequences, likely as a response to prey aggregations near the thermocline. They also showed higher foraging efficiency, performed more dives and dove to shallower depths than those foraging in colder/less‐stratified waters.
4. Reductions in the long‐term memory of dive sequences, or in other words increases in behavioral stochasticity, may suggest different strategies concerning the exploration–exploitation trade‐off under contrasting environmental conditions.

## Graphical abstract

Little penguins foraging in warmer/more‐stratified waters exhibited less stochastic foraging sequences likely as a response to prey aggregations nearby the thermocline. Ultimately, they showed higher foraging efficiency and numbers of dives, and lower mean dive depths than those foraging in colder/less‐stratified waters.

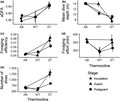

## 1 INTRODUCTION

Understanding the responses and adaptations of organisms to environmental variability has received growing interest in ecology, especially in the context of ongoing global change (Doney et al., 2012). The last IPCC Synthesis Report (IPCC, 2014) noted that marine ecosystems are the most vulnerable to climate change. Climate impacts are already observed from polar to tropical marine ecosystems through, for instance, increases in sea surface temperatures (SST) and altered patterns of ocean climate systems (e.g., El‐Nino Southern Oscillation) (Doney et al., 2012; Ramírez, Afán, Davis, & Chiaradia, 2017). Such changes in physical properties of oceans are associated with changes in marine productivity (Bakun et al., 2015; Polovina, Howell, & Abecassis, 2008), shifts in the distribution and dispersion of organisms (García Molinos et al., 2015), and/or changes in the timing of ecosystem‐level processes (Durant, Hjermann, Ottersen, & Stenseth, 2007). These changes are expected to affect trophic interactions and, by extension, the structure and functioning of ecosystems (Doney et al., 2012). However, due to the complexity of trophic pathways within marine ecosystems, and to the inaccessibility of marine environments, it is extremely difficult to monitor these changes. One approach to addressing these difficulties involves the study of upper‐level predators, which are more sensitive to changes in the marine food chain (Hindell, Bradshaw, Harcourt, & Guinet, 2003; Ramírez et al., 2016).

Among the parameters used to monitor upper‐level predators (e.g., breeding success, populations dynamics), foraging behavior can highlight environmental change at short time scales (Lewis et al., 2006). As upper‐level marine predators forage for resources that are distributed patchily across space and time (Weimerskirch, 2007), they require flexibility in foraging behavior to both meet their energy requirements and provide for their offspring (Weimerskirch, Cherel, Cuenot‐Chaillet, & Ridoux, 1997). These marine predators exhibit variability in foraging behavior in response to variation in sea‐ice distribution (e.g., Bailleul et al., 2007), bathymetry (e.g., Chiaradia, Ropert‐Coudert, Kato, Mattern, & Yorke, 2007), marine currents (e.g., Bost et al., 2009), thermal stratification of the water column (e.g., Takahashi et al., 2008), wind (e.g., Dehnhard, Ludynia, Poisbleau, Demongin, & Quillfeldt, 2013), and SST (e.g., Bost et al., 2015). This variability in environmental features mediates prey availability to predators (Mann & Lazier, 2005), in both prey abundance and distribution in the water column (Boyd et al., 2015; Goundie, Rosen, & Trites, 2015). Understanding how the physical environment and the distribution of prey influence predator activity patterns, and thereby the potential impacts on predator populations, is a research priority in the field of marine megafaunal ecology (Hays et al., 2016).

In that sense, exploring variation in emergent patterns across individuals experiencing different ecological conditions can enhance our understanding of animal–environment interactions (Reynolds, 2015). It has been proposed that complexity in behavior should correspond to complexity in the environment in terms of resource distributions, such that in heterogeneous environments, behavioral sequences are predicted to be more variable and complex, or in other words less deterministic, to enhance prey encounter rates (Alados, Escos, & Emlen, 1996; Humphries, Weimerskirch, Queiroz, Southall, & Sims, 2012; MacIntosh, 2014; MacIntosh, Alados, & Huffman, 2011; Shimada, Minesaki, & Hara, 1995; Viswanathan et al., 1999). For example, we previously showed that variation in the temporal organization of little penguin (*Eudyptula minor*) dive sequences varied across areas with different bathymetric profiles, with penguins foraging in deeper waters producing less deterministic or more stochastic sequences (Meyer et al., 2017). Thus, measuring temporal organization in behavior sequences across environmental gradients can potentially highlight the oceanographic conditions that represent challenging habitats.

In this light, the characterization of fractal properties in animal‐derived time series has emerged as a useful tool to model temporal patterns of animal behavior as a complex process ranging from stochastic, uncorrelated behavior, to deterministic, long‐range dependent (auto‐correlated) behavior that persists across measurement scales (see review in MacIntosh, 2014). The balance between the stochastic and deterministic elements in behavioral sequences may be a critical point in the emergence and maintenance of behavioral flexibility (MacIntosh, 2015; Reynolds, Ropert‐Coudert, Kato, Chiaradia, & MacIntosh, 2015). The degree of complexity in these behaviors is shaped by both internal states (e.g., stress, disease; Alados et al., 1996; Cottin et al., 2014; MacIntosh et al., 2011) and external conditions (e.g., environment; MacIntosh et al., 2011; Meyer et al., 2017). Specific “complexity signatures,” as they have been termed (MacIntosh, 2014), are predicted to optimize the efficiency of biological encounters (Alados et al., 1996; MacIntosh et al., 2011; Shimada et al., 1995). For example, animals might exhibit greater stochasticity in more challenging environments as described above or rely on more ritualized patterns when conditions are amenable to them (MacIntosh et al., 2011; Meyer et al., 2017). Moreover, the competing demands of exploration versus exploitation of resources appear to be a key factor underlying the emergence of fractal patterns in behavior, providing a way of interpreting variation in the scaling properties observed (Reynolds et al., 2015).

Identified as one of the fastest warming marine areas in the world (Hobday & Pecl, 2014; Ramírez et al., 2017; Wu et al., 2012), south‐eastern Australia is experiencing significant changes in both oceanographic features and species distributions (Poloczanska et al., 2007). The region of Bass Strait, located between Tasmania and the Australian mainland, lies at the crossroads of three different marine currents (Sandery & Kämpf, 2005): (a) the warm, nutrient‐poor South Australian Current (SAC); (b) the cold, nutrient‐rich sub‐Antarctic Surface Water (SASW); and (c) the warm, nutrient‐poor East Australian Current (EAC) (see Appendix S1). Changes in the predominance and intensity of one ocean current over another are likely to alter species distributions and thus trophic interactions in the Bass Strait area, as shown previously for the EAC (Oliver et al., 2017; Poloczanska et al., 2007). These changes entrain cascading effects throughout the Bass Strait region's upper‐level predators, such as little penguins (Afán, Chiaradia, Forero, Dann, & Ramírez, 2015; Chiaradia & Nisbet, 2006), Australian fur seals (*Arctocephalus pusillus doriferus*; Hoskins & Arnould, 2014), Australasian gannets (*Morus serrator*; Angel et al., 2015), and short‐tailed shearwaters (*Puffinus tenuirostris*; Berlincourt & Arnould, 2015a).

Among Bass Strait's upper‐level predators, little penguins are useful models for investigating the relationship between environmental variability and complexity in foraging behavior, as it has already been shown that they are sensitive to changes in SST (Berlincourt & Arnould, 2015b; Carroll, Everett, Harcourt, Slip, & Jonsen, 2016), thermal stratification of the water column (Pelletier, Kato, Chiaradia, & Ropert‐Coudert, 2012; Ropert‐Coudert, Kato, & Chiaradia, 2009), and wind (Berlincourt & Arnould, 2015b; Saraux, Chiaradia, Salton, Dann, & Viblanc, 2016). Overall, relatively higher SST, stratified waters, and weak winds are likely favorable for little penguins as they result in higher abundance or predictability of prey patches because prey are aggregated nearby a thermocline (Carroll et al., 2016; Cox, Embling, Hosegood, Votier, & Ingram, 2018). In contrast, lower SST, mixed water layers, or strong winds do not favor such aggregations or abundance, resulting in more challenging conditions for little penguins.

Under these challenging conditions (i.e., low SST, mixed water, and strong winds), we hypothesized that little penguins should exhibit greater stochasticity in the temporal organization of their diving behavior because the more persistent behavioral patterns expected to emerge under less challenging or more homogeneous conditions would be disrupted. Following Reynolds et al. (2015), we hypothesize that foraging sequences may lose their correlation structure if alternations between diving and surface times take on less characteristic or “noisier” patterns, for example, if penguins begin to prioritize exploration, which is freer to vary, overexploitation. To test this hypothesis, we used detrended fluctuation analysis (Peng et al., 1994; Peng, Havlin, Stanley, & Goldberger, 1995) to examine the temporal organization of diving behavior of little penguins in relation to sea surface temperature, water stratification, and wind speeds. We also examined other, more traditional diving variables (i.e., mean dive depth, mean foraging effort per day, foraging efficiency, and mean number of dives per day) in relation to these environmental factors. The study is based on an 11‐year dataset focusing on little penguins at Phillip Island Nature Parks, Australia, and additionally tests for associations between these dive parameters and other variables such as penguin sex (Pelletier, Chiaradia, Kato, & Ropert‐Coudert, 2014) and breeding stage (i.e., incubation, guard, postguard; Chiaradia & Kerry, 1999; Saraux, Robinson‐Laverick, Maho, Ropert‐Coudert, & Chiaradia, 2011).

## 2 METHODS

### 2.1 Long‐term monitoring of foraging behavior

**FIGURE 1:**
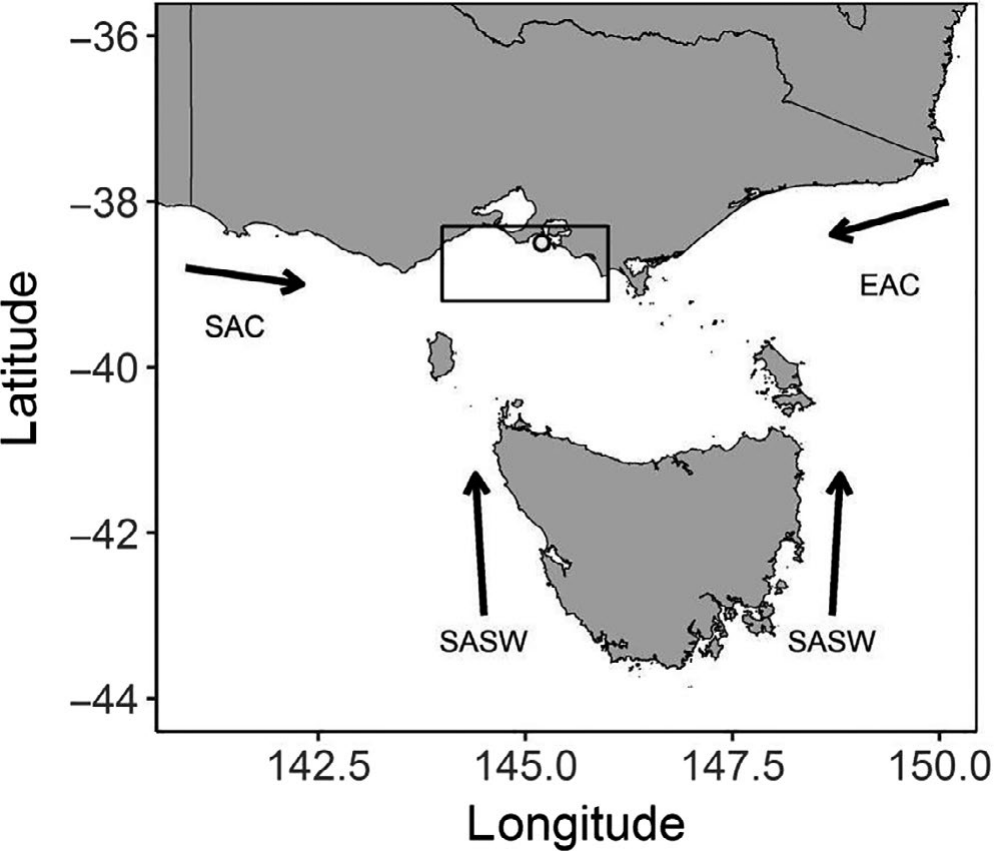
Location of Phillip Island colony (grey dot), foraging area (black rectangle), and a simplified representation of the water masses in south‐eastern Australia (Bass Strait region): South Australian current (SAC), East Australian current (EAC), and Sub‐Antarctic Surface Waters (SASW) (adapted from; Sandery & Kämpf, 2005)

The study was conducted on the little penguin breeding colony at Phillip Island, Australia (38°21′S, 145°09′E, Figure 1), which contains 28,000–32,000 breeding adults (Sutherland & Dann, 2012). We collected diving data from 353 foraging trips over 11 breeding seasons from 2001 to 2012 (except 2003) during the three stages of the breeding cycle, that is, incubation, guard, and postguard. Postguard data were not available for the 2006 season. We used three different types of data‐loggers recording depth at 1 or 2 s intervals (see details in Table 1). Birds were captured inside their nest boxes. Loggers were attached to their lower backs with marine Tesa^®^ tape (Beiersdorf AG, Hamburg, Germany; Wilson et al., 1997). Upon their return from a single trip at sea, birds were recaptured in the colony and loggers were retrieved. Each attachment and removal of the loggers were completed in less than 5 min. All animal research protocol across all years was carried out in accordance with the Phillip Island Nature Parks Animal Experimentation Ethics Committee approval and a research permit issued by the Department of Environment, Land, Water and Planning of the state of Victoria, Australia.

**TABLE 1:** Number of penguins (number of males/females) and logger characteristics for each breeding season

Breeding seasons	2001	2002	2004	2005	2006	2007	2008	2009	2010	2011	2012
Incubation	11 (5/6)	10 (5/5)	10 (6/4)	10 (6/4)	12 (8/4)	9 (5/4)	15 (7/8)	8 (5/3)	10 (5/5)	10 (4/6)	5 (2/3)
Guard	8 (4/4)	12 (6/6)	10 (5/5)	10 (5/5)	10 (5/5)	10 (4/6)	11 (6/5)	8 (5/3)	28 (14/14)	23 (11/12)	10 (5/5)
Postguard	6 (3/3)	15 (7/8)	6 (4/2)	10 (6/4)	–	8 (5/3)	10 (4/6)	11 (5/6)	9 (5/4)	18 (8/10)	10 (5/5)
Loggers	LTD 1200‐100 (Lotek, Canada). 62 × 18 mm^a^, 17 g		M190‐D2GT (Little Leonardo, Japan). 52 × 15 mm^a^, 16 g						ORI400‐D3G (Little Leonardo, Japan). 45 × 12 mm^a^, 9 g		

^a^ The dimensions are given in length and diameter for these cylindrical loggers.

### 2.2 Diving parameters

After recovery, raw data were downloaded from the loggers and analyzed using Igor Pro version 6.22A (Wavemetrics Inc.). We used a purpose‐written script to adjust the depth to zero when birds were at the surface between two dives and extract diving parameters from the depth data, including maximum diving depth, dive and postdive duration, and the number of vertical undulations in the bottom phase of the dive for each dive deeper than 1 m, which are generally indicative of prey pursuit (Kato, Ropert‐Coudert, Grémillet, & Cannell, 2006; Ropert‐Coudert, Kato, Wilson, & Cannell, 2006). A bottom phase was defined as the first and last time during a dive that the depth‐change rate became <0.25 m/s (Kato, Ropert‐Coudert, & Chiaradia, 2008). To allow comparison between the different stages, we calculated the mean foraging effort per day as the total diving duration (in minutes) divided by the trip duration (in days). Similarly, we calculated the mean number of dives per day as the total number of dives performed over a trip divided by the trip duration (in days). The prey encounters per unit time used as proxy for foraging efficiency was calculated as the total number of vertical undulations divided by the total diving duration over the trip (Sala, Wilson, & Quintana, 2012).

### 2.3 Detrended fluctuation analysis

We used Detrended Fluctuation Analysis (DFA; Peng et al., 1994; Peng et al., 1995) to investigate long‐range dependence in the sequential distribution of little penguin dive and postdive durations. DFA is reported to be one of the more robust estimators of the Hurst exponent (Cannon, Percival, Caccia, Raymond, & Bassingthwaighte, 1997), providing a scaling exponent (α_DFA)_ that measures both long‐range correlations and self‐affinity across scales in time series data. Theoretically, α_DFA_ is inversely related to the fractal dimension (Delignières, Torre, & Lemoine, 2005; Eke et al., 2000), which represents an index of structural complexity (Mandelbrot, 1977). This scaling exponent is bound to [0, 1] for fractional Gaussian noises (fGn) and [1, 2] for fractional Brownian motions (fBm) (Delignières et al., 2005; Eke et al., 2000). Values in the range [0.5, 1] and [1.5, 2] reflect persistence while those in the range [0, 0.5] and [1.5, 2] reflect antipersistence in the time series for fGn and fBm, respectively, with 0.5 and 1.5 reflecting randomness (white noise).

Previous studies have shown that dive sequences from foraging penguins are best characterized as persistent, long‐range dependent fractional Gaussian noise (Cottin et al., 2014; Le Guen et al., 2018; MacIntosh, Pelletier, Chiaradia, Kato, & Ropert‐Coudert, 2013; Meyer et al., 2017; Meyer, MacIntosh, Kato, Chiaradia, & Ropert‐Coudert, 2015). In other words, dive and postdive durations of a given length are typically followed by dive and postdive durations of a similar length, with such patterns of fluctuation between these two behavioral states persisting across a range of measurement scales. A figure illustrating the difference in diving sequences between individuals exhibiting high α_DFA_ values and low α_DFA_ values can be found in the supplementary material (Figure S1). We might interpret this as persistent alternations between states of exploration and exploitation that repeat across measurement scales. However, variation in these patterns of fluctuation between states is reflected in variation in scaling exponents derived from DFA: Across penguin species, values can range from 0.75 (more stochastic and less correlated behavior) to 0.99 (heavily deterministic and long‐range dependent/auto‐correlated behavior; MacIntosh et al., unpublished data). Such variation allows characterization of the temporal organization of diving behavior (i.e., foraging complexity) across individuals and conditions (MacIntosh, 2014).

We used two versions of the DFA algorithm in our study. The first (DFA) uses included a linear detrending method in which linear regressions are applied within each window to remove the trends (Peng et al., 1995). We used this algorithm on the first‐order integrated sequences, assuming the original diving data represented an fGn process (MacIntosh et al., 2013). The second (DFA_b_) uses a bridge detrending method in which the first and last points within each window are “bridged” to create a trendline (Cannon et al., 1997). We used this algorithm on the second‐order integrated sequences, which reflect an fBm process (MacIntosh et al., 2013). The outputs of these variants (DFA and DFA_b_), insomuch as they reflect Hurst exponent estimators for fGn and fBm, respectively, are theoretically related to one another through the following relationship: α_fGn_ ≈ α_fBm_ − 1 (Seuront, 2010). Their agreement provides an internal test of validation that the approach is appropriate.

Both methods were run using the package “fractal” (Constantine & Percival, 2014) in R statistical software v3.4.2 (R Development Core Team, 2017). As scaling is generally lacking at the smallest and largest scales, and to avoid any mathematical biases from their inclusion, we used methods provided in (Seuront, 2010) to calculate the best‐scaling regions from which to estimate α_DFA_ and α_DFAb_. A thorough description of this method applied to penguin diving sequence, including DFA calculation, validation of best‐scaling regions, relationships between various fractal dimension estimates and figures are provided in MacIntosh et al. (2013), Cottin et al. (2014), Meyer et al. (2015) and Meyer et al. (2017). As little penguins are visual predators that forage diurnally (Cannell & Cullen, 2008), we only used active diving data (usually from dusk to dawn) and excluded nightly nondiving data. For multiday trips, we calculated α_DFA_ for each day and averaged the daily values over the whole trip.

### 2.4 Environmental variables

The remote‐sensing environmental variables were extracted and averaged over the little penguins' foraging spatial area during the breeding season (approx. 37°45′S–39°38′S, 143°11′E–145°22′E; Collins, Cullen, & Dann, 1999; Hoskins et al., 2008; McCutcheon et al., 2011; Pelletier et al., 2014) unless otherwise specified. For the study period, daily satellite‐derived SST (°C) had a spatial resolution of 0.25° from the National Oceanic and Atmospheric Administration (NOAA) (https://www.esrl.noaa.gov/psd/data/gridded/data.noaa.oisst.v2.highres.html#detail; Reynolds et al., 2007). Daily satellite‐derived wind speed (m/s) had a spatial resolution of 0.25° from NOAA (http://www.ncdc.noaa.gov/data‐access/marineocean‐data/blended‐global/blended‐sea‐winds; Zhang, Bates, & Reynolds, 2006).

**FIGURE 2:**
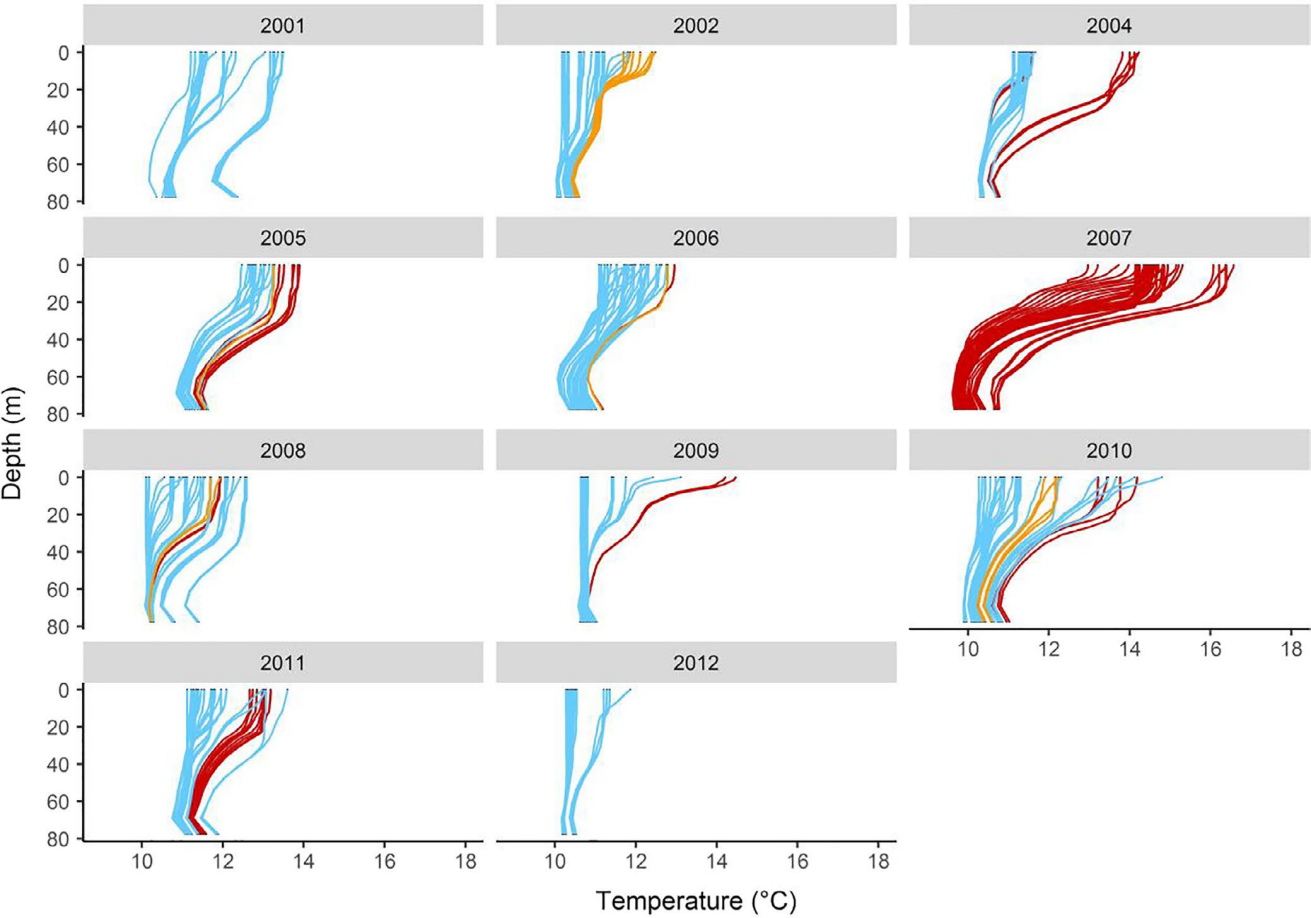
Thermal profiles of the water column in the foraging area of little penguins between the 2001 and 2012 breeding seasons. Each profile corresponds to a day for which a little penguin's trip was recorded. The seabed is situated between 60 m and 80 m. Blue profiles indicate the absence of a thermocline, orange profiles indicate the presence of a weak thermocline, and red profiles indicate the presence of a strong thermocline

Thermal structure in the water column was obtained for the entire study period from a oceanographic simulation model of daily temperature aggregates at 22 depth levels between 0 and 77 m, with a spatial resolution of 0.25°. This dataset was obtained from the Mercator Ocean GLORYS2V4 (1993–2015) global ocean reanalysis for the Global Ocean and Sea Ice Physics project available on the European Union Copernicus Marine Service Information website (http://marine.copernicus.eu/services‐portfolio/access‐to‐products/). These data were also used as a proxy of thermoclines, defined as layers of water in which temperature changes more rapidly with depth than the warm layers above and cold layers below (Fiedler, 2010). To do so, we computed the rate of temperature change with depth (*dT/dD; T*: temperature; *D*:depth) for each vertical temperature profile (Coyle, Pinchuk, Eisner, & Napp, 2008; Takahashi et al., 2008). Based on visual analysis of the graphs (Figure 2) and previous studies in the same area (Ropert‐Coudert et al., 2009), we considered that a thermocline was present in the water column when the highest *dT/dD* exceeded 0.07°C/m. Where thermoclines were detected, we measured the depth of the thermocline, corresponding to the highest *dT/dD*. Based on this depth, we calculated the intensity of the thermocline as the difference between the average water temperature above and below the thermocline (Kokubun et al., 2010). The strength of the thermocline was then used to distinguish between weak (≤1.5°C) and strong (>1.5°C) thermoclines.

### 2.5 Statistics

All statistical analyses were conducted in R v3.4.2 (R Development Core Team, 2017). Preliminary analysis and previous studies (Pelletier, 2013) have shown that SST and water stratification can change concomitantly in the study area. We first tested the effect of SST and wind speed on foraging complexity and diving variables; SST was then substituted by our proxy to the thermal structure of the water column. Models including both SST and thermoclines as predictor variables were not considered because their close correspondence, that is, high SST usually corresponded to strong thermoclines, would lead to considerable variance inflation in the models. Thermocline strength was classified as absent, weak, or strong in the models according to the description above. The distribution of each response variable was checked using the “fitdistrplus” package in R (Delignette‐Muller, Dutang, Pouillot, Denis, & Siberchicot, 2017). We constructed (a) linear mixed‐effects models (LMMs, “lmerTest” package in R; Kuznetsova, Brockhoff, & Christensen, 2016) to investigate variation in α_DFA_ and (b) generalized linear mixed‐effects models (GLMMs, “MASS” package in R; Ripley et al., 2017) to investigate variations in mean dive depth, mean foraging effort per day, foraging efficiency and mean number of dives per day, as a function of SST, wind speed and thermocline presence and strength, as well as bird sex for each stage of the breeding season (detailed results are provided in Appendix S1). As different loggers were used and some individuals may have been sampled more than once across years, logger type and individual were added to the models as random factors. Numeric predictor variables were scaled and centered (z‐transformed) to simplify interpretation of the parameter estimates in the statistical model (Zuur, Ieno, & Smith, 2007). Models were validated by visual examination of histograms of the residuals to ensure homogeneity and plots of residuals versus fitted values to ensure homoscedasticity. Using Cook's distance and visual checking of outliers, we found no evidence of overly influential data points in our models (Fox, 1991). To test for multicollinearity, variance inflation factors (VIF) were calculated for each predictor variable with the package “fmsb” and only variables with values lower than 2 were retained in the models (Zuur, Ieno, & Elphick, 2010). We included a quadratic term for SST if that improved model fit. However, the quadratic term for SST did not explain additional variance in the model so it was removed in favor of better interpretation of the linear effect. All correlation tests were done using the Pearson method. Student's *t* tests were used to compare average SST and wind speed between each stage. Descriptive results are presented as means and standard errors (SE) unless otherwise specified, and we set the alpha level for all statistical analyses at 0.05.

## 3 RESULTS

### 3.1 Fractal analysis of dive sequences

**FIGURE 3:**
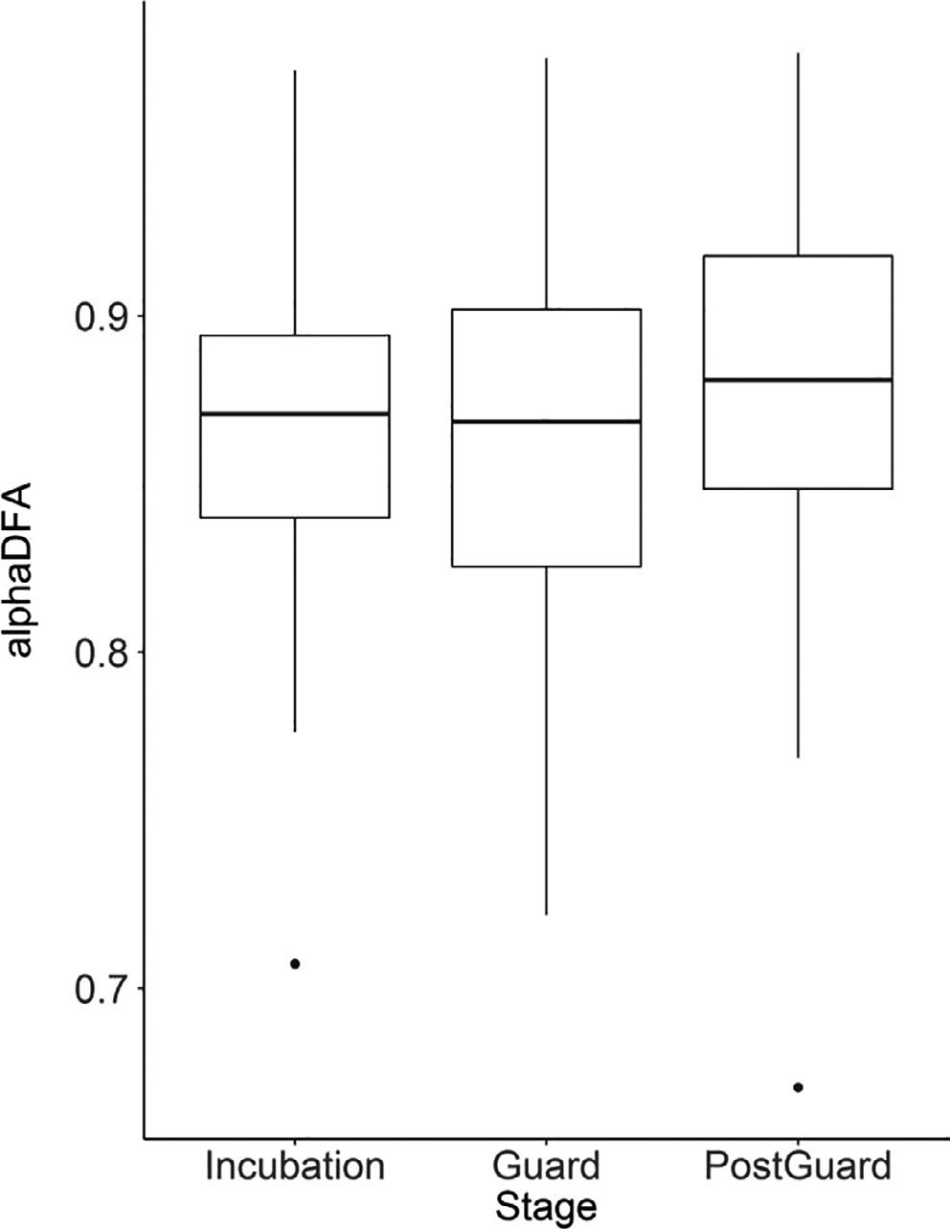
Results of detrended fluctuation analysis (DFA) of diving sequences, showing scaling exponents for each stage

The average scaling exponents observed for penguins foraging during the incubation, guard, and postguard phases were 0.87 ± 0.0004 (α_DFA_) and 1.89 ± 0.004 (α_DFA_b), 0.86 ± 0.0004 and 1.88 ± 0.005, and 0.88 ± 0.0004 and 1.90 ± 0.004, respectively. We observed a strong correlation between the two DFA results at each breeding stage (incubation: *r* = .95, *p* < .001; guard: *r* = .98, *p* < .001; postguard: *r* = .98, *p* < .001). Thus, both methods converged to indicate that dive sequences from foraging little penguins are best characterized as persistent, long‐range dependent fractional Gaussian noise (Figure 3), in accordance with previous studies on penguins (Cottin et al., 2014; Le Guen et al., 2018; MacIntosh et al., 2013; Meyer et al., 2015, 2017). The observed best‐scaling regions were similar for the three stages (incubation, guard and postguard) and included the scales 2^7^–2^11^, or 128–2048 s.

### 3.2 Oceanography

**FIGURE 4:**
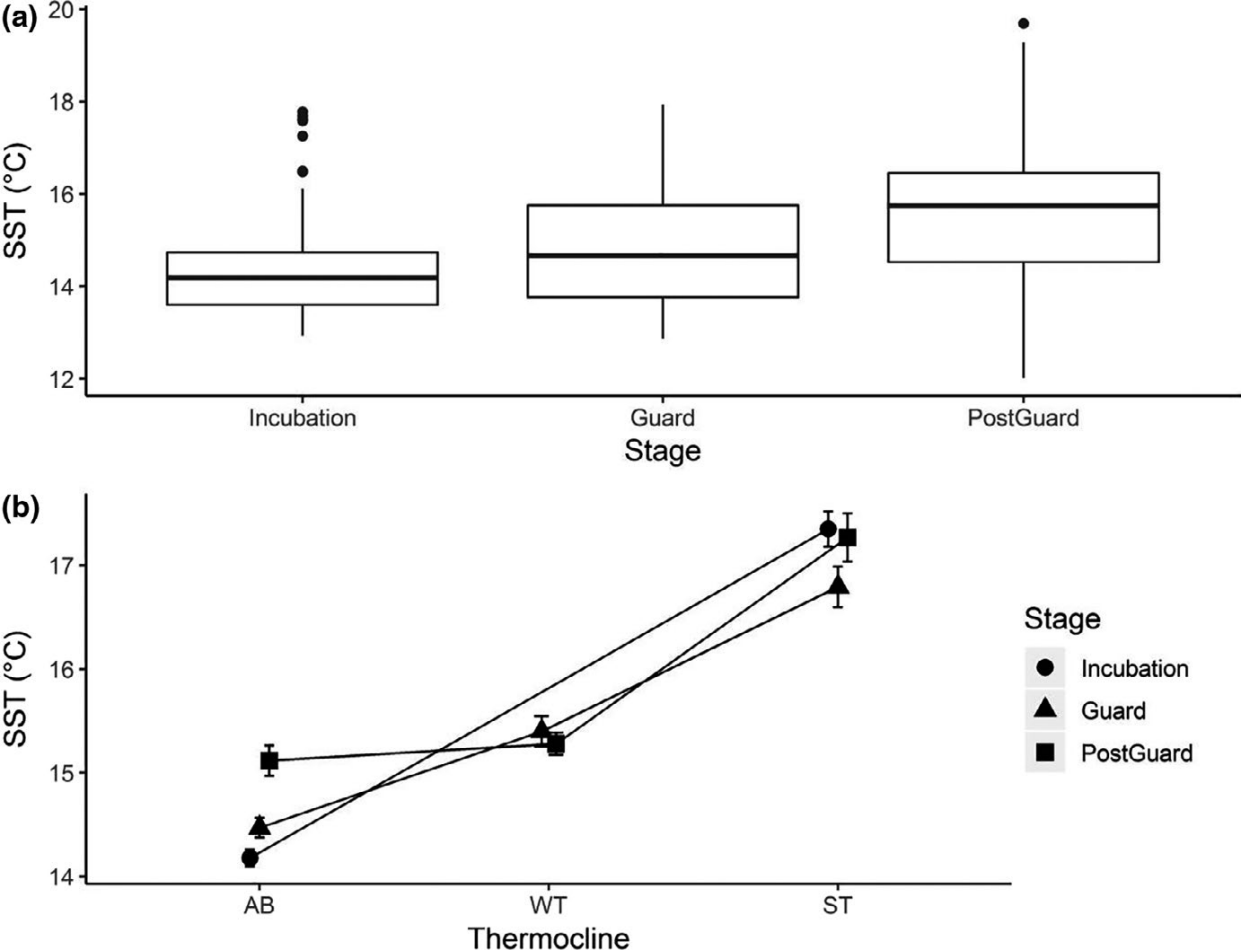
(a) The mean sea surface temperature (2001−2002/2012−2013) for each breeding stage; (b) average sea surface temperature in the absence of a thermocline (AB), the presence of a weak thermocline (WT), and the presence of a strong thermocline (ST) in the water column

As breeding season progress from spring to summer, the mean SST recorded during the foraging trips increased from 14.44°C (*SD* = 1.16°C) to 15.76°C (*SD* = 1.46°C, Student's *t* test: all *p* < .01; Figure 4a). The water column remained well mixed at the beginning of the breeding season, and stratification processes initiated between late October and December concurrently with an increase in SST (Figure 4b). No thermoclines were detected during foraging trips of the 2001 and 2012 breeding seasons. Over the whole study period, only 8% of incubation trips showed the presence of a thermocline, whereas 22% of guard trips and 43% of postguard trips showed the presence of thermoclines. There was no difference in mean wind speed between stages across all years (Student's *t* tests: all *p* > .1). Moreover, no trips coincided with the occurrence of strong winds.

### 3.3 Behavioral organization and environmental parameters

**FIGURE 5:**
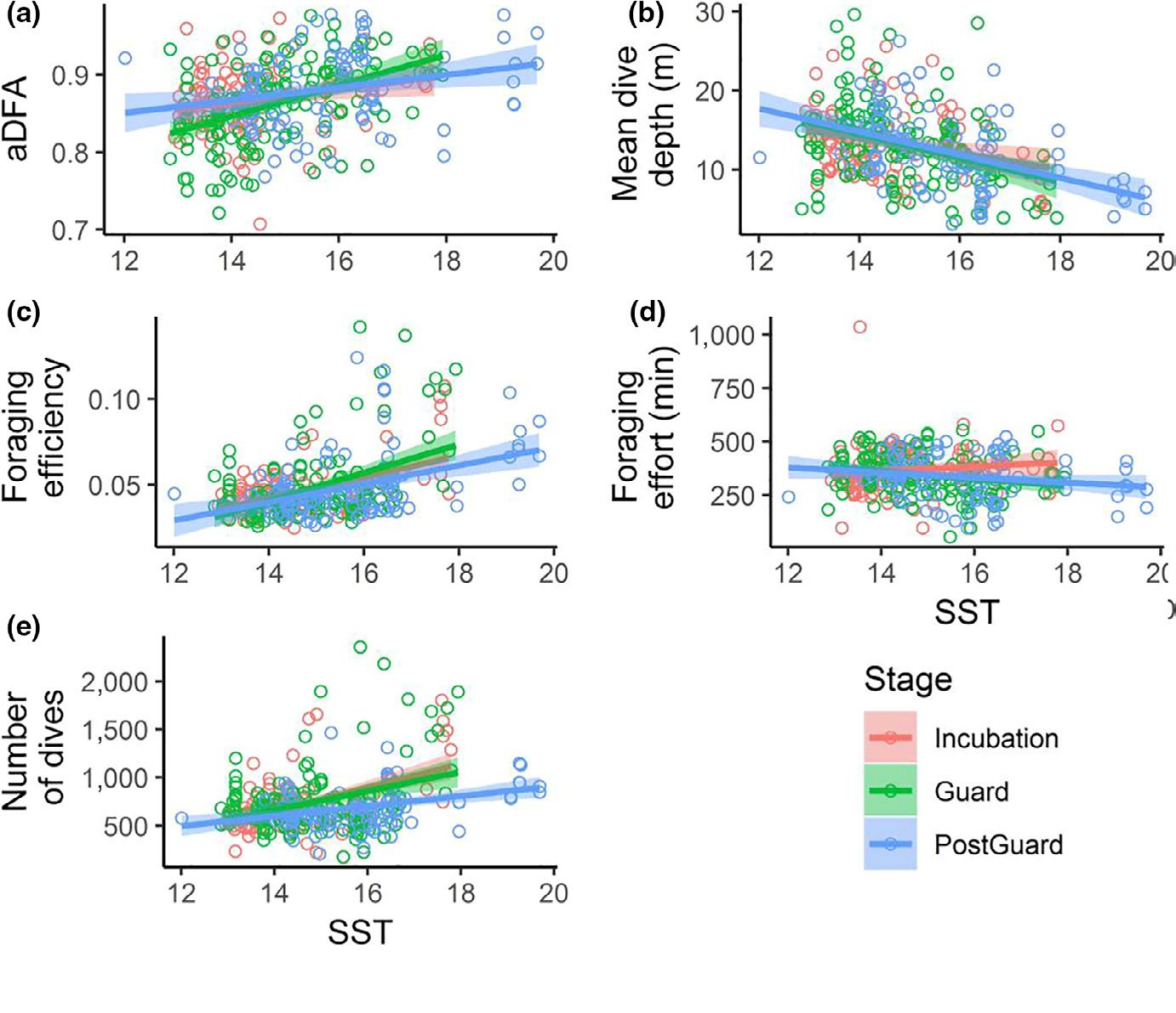
Effects of sea surface temperature (SST in °C) on α_DFA_, mean dive depth (m), foraging efficiency, foraging effort per day, and number of dives per day (Panels a, b, c, d, e). Description of each variable is detailed in the methods. Incubation, guard, postguard stages are, respectively, indicated in red, green, and blue. Each open circle corresponds to one individual value. Each regression line is represented with a 95% confidence interval

**FIGURE 6:**
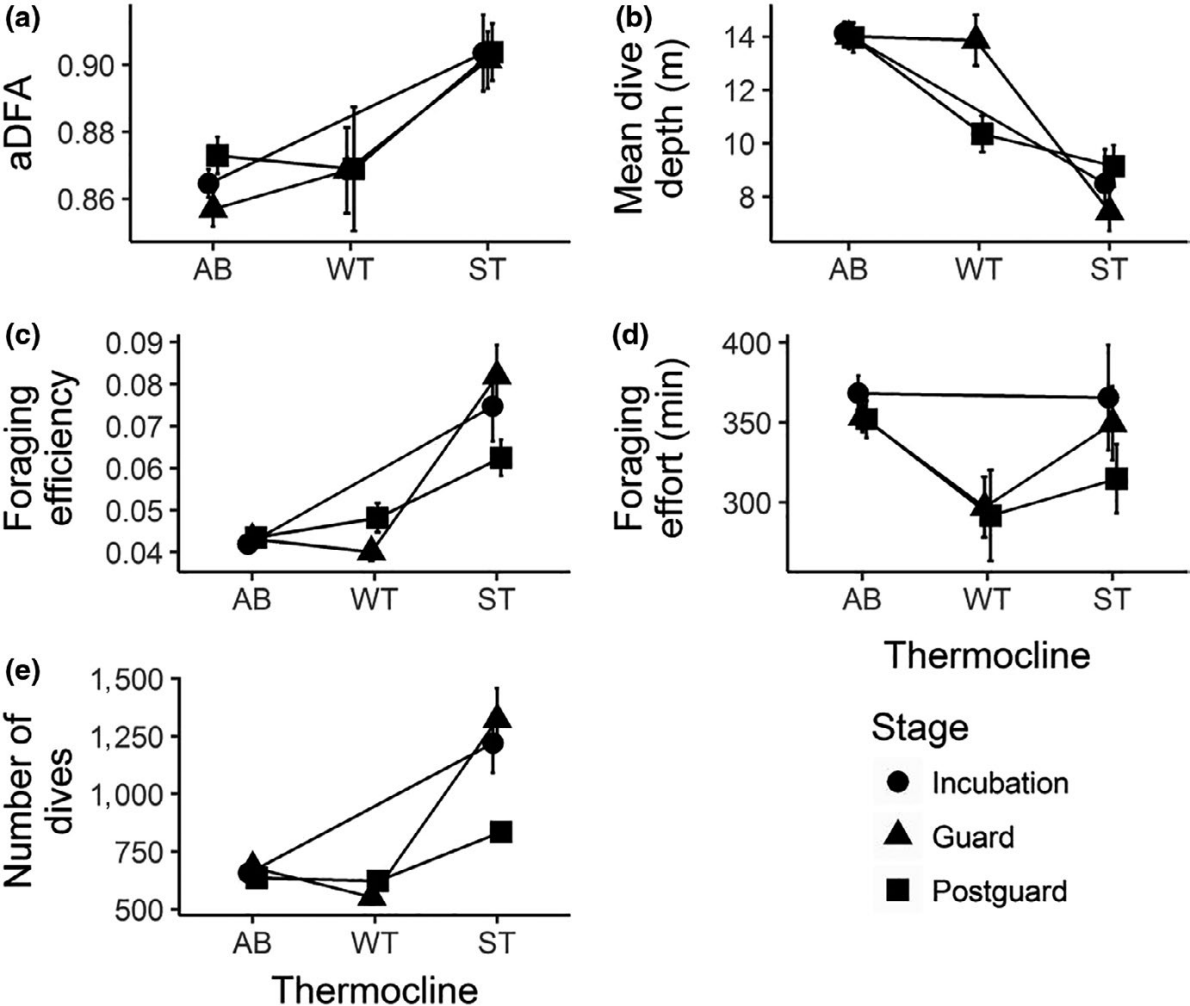
Effects of the absence of a thermocline (AB), the presence of a weak thermocline (WT), and the presence of a strong thermocline (ST) in the water column on α_DFA_, mean dive depth, foraging efficiency, foraging effort per day, and number of dives per day (Panels a, b, c, d, e). Description of each variable is detailed in the methods. Incubation, guard, postguard stages are, respectively, indicated with a black circle, a black triangle, and a black square. Results are presented here as means by thermocline condition and stage with the associated standard errors. No data were collected in presence of weak thermocline (WT) during the incubation stage

Little penguins exhibited higher values of α_DFA_ with higher SST, irrespective of the breeding stage (Table 2; Figure 5a). Higher SST was also linked with shallower dives (Table 2; Figure 5b), higher foraging efficiency (Table 2; Figure 5c), and higher mean numbers of dives per day (Table 1; Figure 5e), again irrespective of breeding stage. Higher SST led to a decrease in mean foraging effort per day only during the postguard stage. At the same time, little penguins exhibited higher values of α_DFA_ (Table 2; Figure 6a), shallower dives (Table 2; Figure 6b), higher foraging efficiency (Table 2; Figure 6c), and larger mean numbers of dives per day (Table 2; Figure 6e) in the presence of stronger thermoclines, irrespective of breeding stage. The presence of a stronger thermocline did not affect mean foraging effort per day, and weak thermoclines were not associated significantly with any aspects of diving behavior observed in this study. Stronger wind speeds were marginally associated with diving variables, as it was only associated with lower values of α_DFA_ during the guard stage for one of the model used (Table 2). Finally, males dived deeper than females during the guard stage, but also showed lower foraging efficiency and lower mean numbers of dives per day than females during incubation (Table 2).

**TABLE 2:** Summary of the effects on diving variables (α_DFA_, mean dive depth, foraging efficiency, foraging effort per day (min), and number of dives per day) of sea surface temperature (SST), wind speed (WS), thermoclines, and sex during the three periods of the breeding season using LMM and GLMM statistics

	Incubation					Guard					Postguard				
Variable	*SST*	WS	*Weak* *Th*	*Strong* *Th*	*Sex M*	*SST*	WS	*Weak* *Th*	*Strong* *Th*	*Sex M*	*SST*	WS	*Weak* *Th*	*Strong* *Th*	*Sex M*
α_DFA_	+	/	/	+	/	+	/−	/	+	/	+	/	/	+	/
Mean dive depth	−	**/**	**/**	**−**	**/**	**−**	**/**	**/**	**−**	**+**	**−**	**/**	**/**	**−**	**/**
Foraging efficiency	+	/	/	+	−	+	/	/	+	/	+	/	/	+	/
Foraging effort/day	/	/	/	/	/	/	/	/	/	/	/	/	/	/	/
Number of dives/day	+	/	/	+	−	+	/	/	+	/	+	/	/	+	/

Note

The complete results are included in Appendix S1. No weak thermocline was recorded during the incubation stage.

Abbreviations: −, negative effect; /, no statistical effect; +, positive effect; Strong Th, strong thermocline; *Weak Th*, weak thermocline.

## 4 DISCUSSION

Sea surface temperature and water stratification influenced the diving behavior of little penguins, in particular the temporal organization of their diving behavior. When exposed to lower SST and less‐stratified waters, little penguins exhibited greater stochasticity in their foraging sequences, but also deeper diving activity, lower foraging efficiency, and smaller numbers of dives, irrespective of breeding stage. These findings suggest that little penguin prey are more dispersed and/or less abundant in colder and less‐stratified waters, leading penguins to spend more time exploring the environment rather than exploiting prey during foraging sequences (Reynolds et al., 2015).

On the one hand, variation in SST is known to influence the abundance and spatial distribution of small pelagic prey. For example, Rhinoceros auklets (*Cerorhinca monocerata*) breeding in the Sea of Japan are highly dependent on the seasonal availability of Japanese anchovy (*Engraulis japonicas*), which is positively related to increases in SST (Takahashi et al., 2001). Similarly, the optimal offshore temperature range for little penguin prey–capture success (Carroll et al., 2016) seems to mirror the thermal peak of availability of sardines (*Sardinops sagax*; Agenbag et al., 2003; Nevárez‐Martínez et al., 2001). In our study, the maximum SST was in the optimal offshore temperature range for capture success observed by Carroll et al. (2016), so that the positive association between foraging parameters and increasing SST (i.e., more deterministic sequences, shallower dives, higher foraging efficiency and higher numbers of dives) can reflect an improvement in foraging conditions for these penguins (e.g., higher prey availability), although the situation could become detrimental to foraging success if SST continues to increase and move away from the optimal temperature noted by Carroll et al. (2016).

On the other hand, strong water stratification, an indication of the presence of a thermocline in the water column, could enhance prey availability in the foraging area by acting as a physical barrier to prey dispersion, creating an aggregation of marine life (McInnes, Ryan, et al., 2017; Ropert‐Coudert et al., 2009). Here, with strong thermoclines in the water column, penguins showed more determinism (less stochasticity) in their foraging sequences, as well as greater foraging efficiency, shallower dives, and higher numbers of dives, than they did in the presence of weak thermoclines or in their absence altogether. Interestingly, it has been shown that penguins foraging in shallower environments also exhibited greater determinism in their foraging sequences, as well as greater foraging efficiency and shallower dives, when compared to individuals foraging in deeper waters (Meyer et al., 2017). The potential for thermoclines to act as a physical barrier to prey, and thereby mimic the influence of bathymetric structure on both prey distribution and diving behavior, is also consistent with previous results concerning the role of the thermoclines/water stratification in prey encounters (Boyd et al., 2015; Kokubun et al., 2010; Pelletier et al., 2012; Ropert‐Coudert et al., 2009; Waggitt et al., 2018).

Our results suggest that both higher SST and the presence of thermoclines lead to increased prey availability in the foraging area, which is reflected in the higher foraging performance of little penguins under such favorable environmental conditions. Moreover, such situations are likely to occur toward the second half of the breeding season, in particular during the chick‐rearing (guard and postguard) stages. Both of these stages are critical periods for the growth and development of chicks, so changes in foraging efficiency at this stage may have dramatic consequences on reproductive output (Chiaradia & Nisbet, 2006). Future studies should investigate how the absence/presence of thermoclines in the foraging area over a season may influence the breeding success at the colony level.

The link between the temporal organization of the diving behavior and thermocline strength seems to reflect a trade‐off pitting exploration of the environment against exploitation of prey as previously proposed (Reynolds et al., 2015). Under this exploration/exploitation trade‐off, the greater stochasticity in sequences of foraging behavior in colder and less‐stratified waters resulted in an increase in target depths or variance from one dive to the next, which is likely to enhance information gathering about prey locations with increasingly heterogeneous prey fields. This pattern would lead to deeper dives on average and induce variability in both dive and postdive durations, reducing long‐range dependence in dive sequences and thus leading to greater stochasticity in behavior.

In this context, when exploration of the environment is favored, foraging efficiency and numbers of dives decrease. Conversely, warmer and strongly‐stratified waters favor exploitation due to increased accessibility and predictability of prey (Kokubun et al., 2010; Pelletier et al., 2012; Ropert‐Coudert et al., 2009). Under favorable conditions, individuals may favor exploitation over exploration, leading to greater determinism in sequences of foraging behavior but also shallower dives, greater foraging efficiency and smaller overall numbers of dives performed. This more persistent and periodic behavior in periods of prey exploitation could reflect the physiological limits that constrain both dive duration and postdive duration, such as oxygen reserves (Wilson, 2003) and lactic acid build‐up (Butler, 2006). Under an environment of high prey availability at shallower depths, individuals could dedicate more significant amounts of time to the bottom phase of dives, wherein feeding events are likely to happen (Ropert‐Coudert et al., 2006), ultimately increasing foraging efficiency.

Despite having a significant effect on some foraging parameters of little penguins (e.g., foraging effort, trip duration; Berlincourt & Arnould, 2015b; Saraux et al., 2016), wind speed had only a marginal effect on diving behavior in our study. Stronger wind speed was only associated with higher stochasticity in foraging sequences (for only one model) in guard stage. Wind are known to cause mixing of the water column, modifying the stratification of the waters (Acha, Mianzan, Guerrero, Favero, & Bava, 2003) and by so the predictability of prey fields. This is consistent with the other results described in this study in which penguins exhibited higher stochasticity in foraging sequences in response to challenging environmental conditions. However, we only detected such effect during the guard stage and it could be explained by the fact we did not record extreme wind speeds (>14 m/s), representative of storm conditions (Dehnhard et al., 2013; Saraux et al., 2016), during this study.

Using a comprehensive dataset over an 11‐year period, we showed how the temporal organization of the diving behavior in a seabird might vary according to intra‐ and interannual variability of environmental conditions. More stochastic foraging sequences may reflect more challenging environmental conditions (e.g., colder and less‐stratified waters), where prey patches are less predictable and when a switch toward more exploratory behavior is needed to buffer such changes. This approach might aid future studies aiming to understand how animal movement is affected by the distribution of prey and the physical environment. One component these studies could also take into account is how individual and social environment characteristics, especially for group‐hunting species such as penguins (Berlincourt & Arnould, 2014; McInnes, McGeorge, Ginsberg, Pichegru, & Pistorius, 2017), shape the temporal organization of the diving behavior in marine predators, and their decisions to explore or exploit the environment.

## CONFLICT OF INTEREST

None declared.

## AUTHOR CONTRIBUTIONS

**Xavier Meyer:** Conceptualization (equal); data curation (supporting); formal analysis (lead); writing–original draft (lead); writing–review and editing (lead). **Andrew J. J. MacIntosh:** Conceptualization (equal); formal analysis (supporting); supervision (equal); writing–review and editing (supporting). **Andre Chiaradia:** Data curation (lead); writing–review and editing (supporting). **Akiko Kato:** Data curation (lead); writing–review and editing (supporting). **Francisco Ramírez:** Data curation (lead); writing–review and editing (supporting). **Cedric Sueur:** Supervision (lead); writing–review and editing (supporting). **Yan Ropert‐Coudert:** Conceptualization (equal); supervision (lead); writing–review and editing (supporting).

## Supporting information

Supplementary Material

## Data Availability

Diving data and metadata are archived and available on Dryad (https://doi.org/10.5061/dryad.6hdr7sqxk).
