# Supplementary material for: Oceanic thermal structure mediates dive sequences in a foraging seabird
Source: Ecol Evol. 2020 May 24;10(13):6610–22. doi: 10.1002/ece3.6393 (PMC7381582; doi:10.1002/ece3.6393)
Supplement: Supplementary file 1 — Supplementary Material [file ECE3-10-6610-s001.docx]

Supporting Information for:

**Oceanic thermal structure mediates dive sequences in a foraging seabird**

**Xavier Meyer^a,b*^, Andrew J.J. MacIntosh^b^, Andre Chiaradia^c^, Akiko Kato^d^, Francisco Ramírez^e^, Cédric Sueur^a^ & Yan Ropert-Coudert^d^**

^a^Université de Strasbourg, CNRS, IPHC UMR7178, F-67000 Strasbourg, France, ^b^Kyoto University Primate Research Institute, Kanrin 41-2, Inuyama, Aichi 484-8506, Japan, ^c^Phillip Island Nature Parks, Conservation Department, P.O. Box 97, Cowes, Victoria 3922, Australia, ^d^Centre d'Etudes Biologiques de Chizé, CNRS UMR 7372 - Université de La Rochelle, 79360 Villiers-en-Bois, France, ^e^Departament de Biologia Evolutiva, Ecologia i Ciènces Ambientals, Universitat de Barcelona, Av. Diagonal 643, 08028, Barcelona, Spain.

*Correspondence should be addressed to X.M., Kyoto University Primate Research Institute, Kanrin 41-2, Inuyama, Aichi, 484-8506, Japan (email: xavier.g.meyer@gmail.com).

**Detrended Fluctuation Analysis**

**
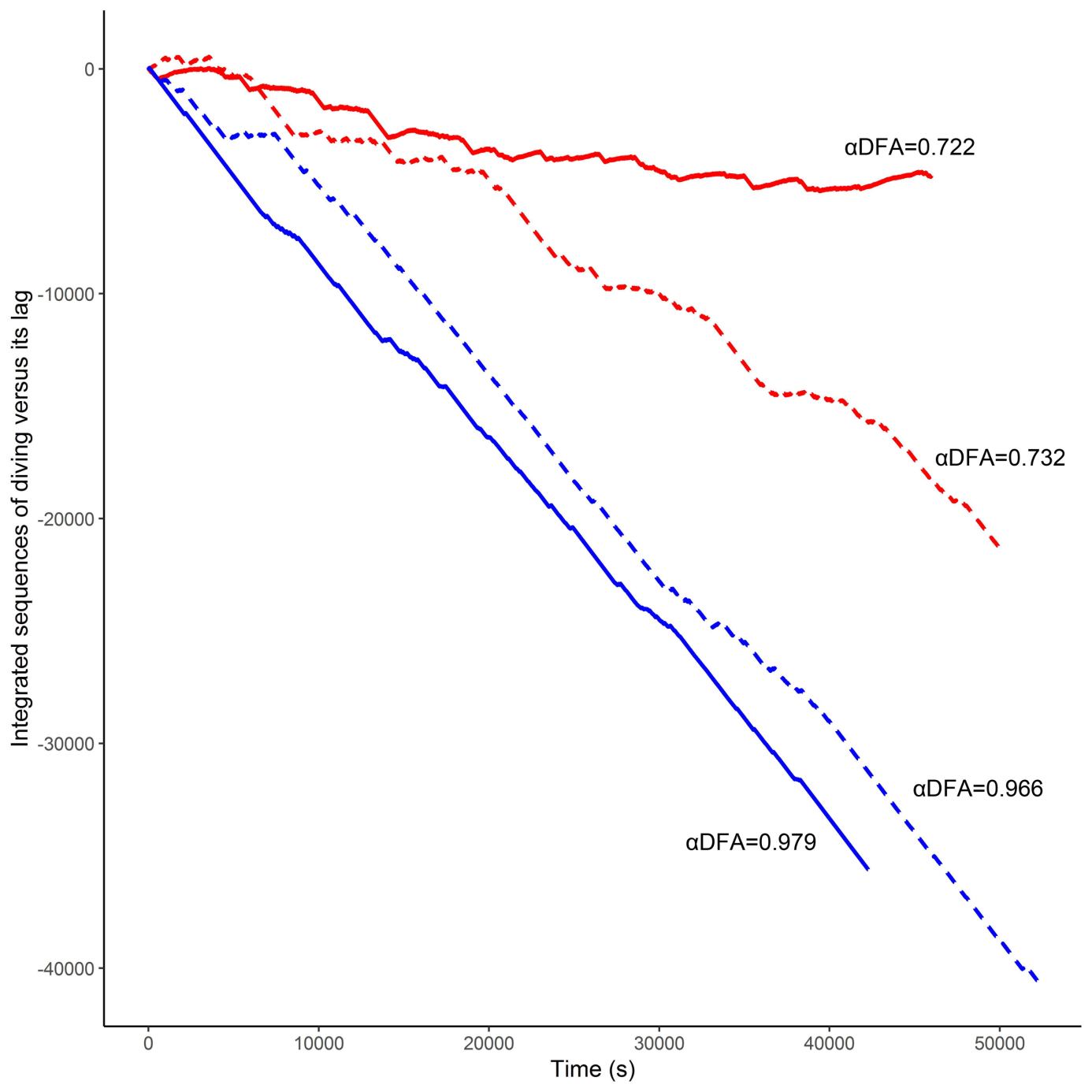
**

Figure S1: Example of integrative (cumulatively summed) dive sequences of 4 different little penguins showing variations in foraging patterns and resultant changes in α_DFA_ values. The blue lines indicate the integrated diving sequences of two little penguins exhibiting high α_DFA_ values and the red lines indicated the integrated diving sequences of two little penguins exhibiting low α_DFA_ values.

**Results**

Table S2: Effects on α_DFA_ of sea surface temperature (SST), wind speed (WS), thermoclines and sex during the three periods of the breeding season using LMM statistics. No weak thermocline was recorded during the incubation stage. Bold text highlights significant effects. Abbreviations: *β* =estimate, *SE*= Standard error.

|  | Incubation | | | | Guard | | | | Post-guard | | | |
| --- | --- | --- | --- | --- | --- | --- | --- | --- | --- | --- | --- | --- |
| Variable | *β* | SE | *t-value* | *p-value* | *β* | SE | *t-value* | *p-value* | *β* | SE | *t-value* | *p-value* |
| Intercept | 0.744 | 0.053 | 13.978 | <0.001 | 0.588 | 0.057 | 10.229 | <0.001 | 0.747 | 0.059 | 12.579 | <0.001 |
| SST | **0.009** | **0.003** | **2.550** | **0.014** | **0.019** | **0.003** | **5.617** | **<0.001** | **0.008** | **0.003** | **2.468** | **0.015** |
| WS | -0.0004 | 0.002 | -0.208 | 0.835 | -0.001 | 0.002 | -0.808 | 0.420 | 0.0008 | 0.002 | 0.386 | 0.700 |
| SexM | -0.002 | 0.008 | -0.240 | 0.811 | -0.003 | 0.008 | -0.434 | 0.665 | 0.001 | 0.009 | 0.130 | 0.897 |
| Intercept | 0.871 | 0.017 | 51.198 | <0.001 | 0.892 | 0.018 | 50.569 | <0.001 | 0.886 | 0.019 | 45.396 | <0.001 |
| Weak Thermocline | - | - | - | - | 0.010 | 0.014 | 0.725 | 0.469 | -0.016 | 0.014 | -1.174 | 0.243 |
| Strong  Thermocline | **0.04** | **0.015** | **2.801** | **0.006** | **0.054** | **0.014** | **3.807** | **<0.001** | **0.038** | **0.011** | **3.471** | **<0.001** |
| WS | -0.0007 | 0.002 | -0.371 | 0.711 | **-0.004** | **0.002** | **-2.373** | **0.019** | -0.001 | 0.002 | -0.651 | 0.516 |
| SexM | -0.002 | 0.008 | -0.271 | 0.787 | -0.003 | 0.009 | -0.401 | 0.689 | 0.0004 | 0.009 | 0.040 | 0.968 |

Table S3: Effects on mean dive depth of sea surface temperature (SST), wind speed (WS), thermocline and sex during the three periods of the breeding season using GLMM statistics. Bold text highlights significant effects. Abbreviations: *β* =estimate, *SE*= Standard error.

|  | Incubation | | | | Guard | | | | Post-guard | | | |
| --- | --- | --- | --- | --- | --- | --- | --- | --- | --- | --- | --- | --- |
| Variable | *β* | SE | *t-value* | *p-value* | *β* | SE | *t-value* | *p-value* | *β* | SE | *t-value* | *p-value* |
| Intercept | 3.488 | 0.431 | 8.085 | <0.001 | 4.729 | 0.421 | 11.237 | <0.001 | 4.283 | 0.433 | 9.885 | <0.001 |
| SST | **-0.071** | **0.028** | **-2.504** | **0.029** | **-0.136** | **0.026** | **-5.265** | **<0.001** | **-0.115** | **0.025** | **-4.574** | **0.001** |
| WS | 0.015 | 0.015 | 0.996 | 0.341 | -0.026 | 0.013 | -2.039 | 0.058 | 0.007 | 0.015 | 0.438 | 0.671 |
| SexM | 0.071 | 0.060 | 1.179 | 0.263 | **0.170** | **0.061** | **2.770** | **0.014** | -0.050 | 0.070 | -0.714 | 0.477 |
| Intercept | 2.494 | 0.121 | 20.568 | <0.001 | 2.629 | 0.122 | 21.563 | <0.001 | 2.426 | 0.135 | 17.987 | <0.001 |
| Weak Thermocline | - | - | - | - | -0.11 | 0.090 | -1.227 | 0.239 | -0.070 | 0.099 | -0.703 | 0.499 |
| Strong Thermocline | **-0.524** | **0.135** | **-3.873** | **0.003** | **-0.730** | **0.130** | **-5.610** | **<0.001** | **-0.363** | **0.083** | **-4.363** | **0.002** |
| WS | 0.016 | 0.015 | 1.127 | 0.283 | -0.004 | 0.012 | -0.337 | 0.741 | 0.026 | 0.015 | 1.677 | 0.128 |
| SexM | 0.084 | 0.058 | 1.442 | 0.177 | **0.152** | **0.059** | **2.600** | **0.020** | -0.035 | 0.070 | -0.504 | 0.615 |

Table S4: Effects on foraging efficiency of sea surface temperature (SST), wind speed (WS), thermocline and sex during the three periods of the breeding season using GLMM statistics. Bold text highlights significant effects. Abbreviations: *β* =estimate, *SE*= Standard error.

|  | Incubation | | | | Guard | | | | Post-guard | | | |
| --- | --- | --- | --- | --- | --- | --- | --- | --- | --- | --- | --- | --- |
| Variable | *β* | SE | *t-value* | *p-value* | *β* | SE | *t-value* | *p-value* | *β* | SE | *t-value* | *p-value* |
| Intercept | -5.055 | 0.277 | -18.262 | <0.001 | -5.948 | 0.328 | -18.105 | <0.001 | -4.298 | 0.439 | -9.792 | <0.001 |
| SST | **0.146** | **0.018** | **8.159** | **<0.001** | **0.188** | **0.020** | **9.466** | **<0.001** | **0.087** | **0.024** | **2.666** | **0.004** |
| WS | -0.017 | 0.013 | -1.265 | 0.232 | 0.015 | 0.011 | 1.401 | 0.180 | -0.012 | 0.015 | -0.775 | 0.456 |
| SexM | **-0.109** | **0.050** | **-2.189** | **0.051** | -0.114 | 0.061 | -1.873 | 0.079 | -0.010 | 0.072 | -0.141 | 0.888 |
| Intercept | -2.976 | 0.093 | -31.998 | <0.001 | -2.999 | 0.103 | -29.176 | <0.001 | -2.850 | 0.127 | -22.376 | <0.001 |
| Weak Thermocline | - | - | - | - | -0.050 | 0.101 | -0.499 | 0.625 | -0.043 | 0.110 | -0.390 | 0.705 |
| Strong Thermocline | **0.676** | **0.060** | **11.296** | **<0.001** | **0.686** | **0.076** | **8.955** | **<0.001** | **0.284** | **0.070** | **4.046** | **0.003** |
| WS | -0.020 | 0.012 | -1.697 | 0.118 | -0.014 | 0.011 | -1.253 | 0.229 | -0.030 | 0.014 | -2.085 | 0.067 |
| SexM | **-0.115** | **0.045** | **-2.550** | **0.027** | -0.083 | 0.059 | -1.401 | 0.181 | -0.004 | 0.071 | -0.053 | 0.957 |

Table S5: Effects on foraging effort per day (min) of sea surface temperature (SST), wind speed (WS), thermocline and sex during the three periods of the breeding season using GLMM statistics. Bold text highlights significant effects. Abbreviations: *β* =estimate, *SE*= Standard error.

|  | Incubation | | | | Guard | | | | Post-guard | | | |
| --- | --- | --- | --- | --- | --- | --- | --- | --- | --- | --- | --- | --- |
| Variable | *β* | SE | *t-value* | *p-value* | *β* | SE | *t-value* | *p-value* | *β* | SE | *t-value* | *p-value* |
| Intercept | 5.426 | 0.351 | 15.439 | <0.001 | 546.973 | 106.320 | 5.145 | <0.001 | 422.682 | 128.134 | 3.299 | <0.001 |
| SST | 0.027 | 0.023 | 1.179 | 0.263 | -12.929 | 6.269 | -2.062 | 0.056 | -9.595 | 7.137 | -1.344 | 0.208 |
| WS | 0.017 | 0.014 | 1.170 | 0.267 | 0.580 | 3.280 | 0.177 | 0.862 | 7.120 | 4.616 | 1.560 | 0.150 |
| SexM | -0.063 | 0.055 | -1.146 | 0.276 | 8.641 | 14.558 | 0.593 | 0.561 | 12.277 | 20.189 | 0.608 | 0.545 |
| Intercept | 5.818 | 0.118 | 49.372 | <0.001 | 342.955 | 37.507 | 9.144 | <0.001 | 270.686 | 39.323 | 6.884 | <0.001 |
| Weak Thermocline | - | - | - | - | -37.875 | 24.862 | -1.523 | 0.148 | -24.268 | 29.570 | -0.821 | 0.433 |
| Strong Thermocline | -0.009 | 0.102 | -0.091 | 0.929 | 2.229 | 24.995 | 0.089 | 0.930 | -46.702 | 23.303 | -2.004 | 0.076 |
| WS | 0.016 | 0.014 | 1.135 | 0.281 | 2.976 | 3.445 | 0.863 | 0.401 | 9.240 | 4.385 | 2.107 | 0.064 |
| SexM | -0.063 | 0.056 | -1.131 | 0.282 | 11.234 | 14.843 | 0.757 | 0.461 | 14.764 | 19.969 | 0.739 | 0.462 |

Table S6: Effects on the number of dives per day of sea surface temperature (SST), wind speed (WS), thermocline and sex during the three periods of the breeding season using GLMM statistics. Bold text highlights significant effects. Abbreviations: *β* =estimate, *SE*= Standard error.

|  | Incubation | | | | Guard | | | | Post-guard | | | |
| --- | --- | --- | --- | --- | --- | --- | --- | --- | --- | --- | --- | --- |
| Variable | *β* | SE | *t-value* | *p-value* | *β* | SE | *t-value* | *p-value* | *β* | SE | *t-value* | *p-value* |
| Intercept | 4.199 | 0.334 | 12.571 | <0.001 | 4.203 | 0.496 | 8.471 | <0.001 | 5.148 | 0.360 | 14.289 | <0.001 |
| SST | **0.166** | **0.021** | **7.854** | **<0.001** | **0.155** | **0.029** | **5.276** | **<0.001** | **0.081** | **0.019** | **4.204** | **0.002** |
| WS | 0.002 | 0.017 | 0.137 | 0.893 | 0.018 | 0.016 | 1.064 | 0.303 | 0.012 | 0.013 | 0.920 | 0.379 |
| SexM | **-0.147** | **0.061** | **-2.403** | **0.035** | -0.095 | 0.076 | -1.249 | 0.230 | 0.044 | 0.055 | 0.801 | 0.425 |
| Intercept | 6.565 | 0.130 | 50.500 | <0.001 | 6.655 | 0.121 | 54.786 | <0.001 | 6.513 | 0.101 | 64.487 | <0.001 |
| Weak Thermocline | - | - | - | - | -0.209 | 0.147 | -1.426 | 0.171 | -0.085 | 0.093 | -0.915 | 0.384 |
| Strong Thermocline | **0.622** | **0.071** | **8.760** | **<0.001** | **0.662** | **0.068** | **9.786** | **<0.001** | **0.254** | **0.057** | **4.463** | **0.002** |
| WS | 0.0002 | 0.016 | 0.010 | 0.992 | -0.012 | 0.014 | -0.839 | 0.414 | -0.008 | 0.011 | -0.712 | 0.494 |
| SexM | **-0.154** | **0.060** | **-2.558** | **0.027** | -0.084 | 0.064 | -1.304 | 0.212 | 0.042 | 0.054 | 0.786 | 0.434 |
